# Unveiling the anti-inflammatory power of star anise-cinnamon compound essential oil against MDR *Salmonella*

**DOI:** 10.3389/fcimb.2026.1814361

**Published:** 2026-07-13

**Authors:** Ziheng Xu, Jie Zhang, Rongdian Lin, Zuxiang Luan, Kai Shao, Yanhua Chen, Jie Liang, Chuangrong Huang, Dapei Zhang, Junyu Tao, Haibo Tang, Weizhong Huang, Longjian Huang, Hai Li, Dingqiang Chen

**Affiliations:** 1 Zhujiang Hospital of Southern Medical University, Guangzhou, China; 2 Postdoctoral Innovation Base of Southern Medical University, Guangdong Luofushan Tranditional Medicine Co., LTD., Huizhou, China; 3 School of Public Health and Management, Guangxi University of Chinese Medicine, Nanning, China; 4 Guangxi Key Laboratory of Translational Medicine for Treating High-incidence Infectious with Integrative Medicine, Guangxi University of Chinese Medicine, Nanning, China; 5 Student Employment Center, Nanning Normal University, Nanning, China; 6 Headmaster’s Office, Youjiang Medical University for Nationalities, Baise, China

**Keywords:** cinnamon, essential oil, multidrug resistance, network pharmacology and molecular docking, *Salmonella Thompson*, star anise, TLR4/NF-κB inflammatory pathway

## Abstract

**Introduction:**

This study aimed to investigate the antiinfi ammatory effects and potential molecular mechanisms of star anise-cinnamon compound essential oil (SCEO) against multidrug-resistant Salmonella Thompson (MDR S. Thompson) infection in mice.

**Methods:**

An intestinal infection model was established in mice by oral gavage of MDR S. Thompson (1×109 CFU/mL) for two consecutive days. Experimental animals were randomly assigned to a blank group, a model group, a levofioxacin group (positive control), and SCEO low-, medium-, and high-dose groups (100, 200, and 400mg/kg, respectively).

**Results and Discussion:**

SCEO intervention significantly alleviated clinical symptoms in infected mice, restored pathological damage in the liver and ileum, and normalized the liver index. Gas chromatography-mass spectrom- etry (GC-MS) analysis identified trans-cinnamaldehyde and trans-anethole as the primary active components of SCEO. Network pharmacology and molecular docking analyses revealed strong binding affinities between these components and Toll-like receptor 4 (TLR4), RELA, and mitogen-activated protein kinase 14 (MAPK14) (Vina score < -5.0 kcal/mol). Further in vivo and in vitro experiments demonstrated that SCEO significantly downregulated the mRNA expression of key cytokines, including Tlr4, interleukin-6 (Il6), tumor necrosis factor (Tnf), and interleukin-17A (Il17a), and suppressed the overactivation of the TLR4/nuclear factor kappa-B (NFĸB)/MAPK signaling pathway. SCEO effectively alleviated Salmonella-induced infiammatory damage, a protective process potentially me- diated through the modulation of the TLR4/NFĸB/MAPK signaling axis. These findings provide a theoretical basis for the development of SCEO as a natural, plant-derived anti-infective agent.

## 1 Introduction

Bacterial food-borne diseases remain a significant global public health concern. In China, bacterial pathogens account for 51.5% of food-borne disease outbreaks, with Salmonella identified as the leading causative agent (Fu et al., 2019; He et al., 2023). The widespread misuse of antibiotics and environmental disinfectants has driven bacterial evolution, leading to a sharp increase in multidrug-resistant (MDR) *Salmonella* strains (Darby et al., 2023; Wu-Chen et al., 2023). Since 2010, *Salmonella* resistance rates to commonly used clinical antibiotics, such as quinolones and β-lactams, have risen significantly (Wang et al., 2023; Xu et al., 2021). The emergence of highly virulent MDR *Salmonella* not only elevates the risk of severe food-borne illness but also significantly compromises the effectiveness of conventional antimicrobial therapies, underscoring the urgent need for alternative intervention strategies.

Unlike single-target antibiotics, Traditional Chinese Medicine (TCM) typically employs multi-component formulations that act on multiple targets, which may reduce the likelihood of resistance development. Among TCM-derived agents, essential oils from cinnamon (*Cinnamomum cassia*) and star anise (*Illicium verum*) have been investigated for their antimicrobial and anti-inflammatory activities. *Trans*-cinnamaldehyde, the major active constituent of cinnamon essential oil, has been reported to inhibit *Salmonella* adhesion to host cells by suppressing type I pili expression, while also modulating oxidative stress and inflammatory responses (Yin et al., 2022a, Yin et al., 2022b; Zhao et al., 2023). Star anise essential oil similarly exhibits antimicrobial activity against a range of pathogenic microorganisms (Saraswathy et al., 2010). Moreover, combinations of essential oils may exert synergistic antimicrobial and anti-inflammatory effects, thereby enhancing overall efficacy (Huang et al., 2024).

Recent advances in oil formulation technologies, including nanoemulsion-based delivery systems and ozonation, have significantly improved the stability, bioavailability, and biological performance of plant-derived oils (Al-Rajhi and Abdel Ghany, 2023; Al-Rajhi et al., 2025a, Al-Rajhi et al., 2025b). In parallel, molecular docking has emerged as a valuable in silico approach for predicting interactions between oil-derived bioactive compounds and their potential targets, thereby enabling mechanistic insights (Al Abboud et al., 2024; Al-Rajhi et al., 2025a, Al-Rajhi et al., 2025b). However, despite these advances in formulation optimization and target prediction, a more comprehensive understanding of the *in vivo* anti-inflammatory mechanisms underlying the synergistic effects of combined essential oils against multidrug-resistant enteric pathogens is still needed.

Building upon our previous *in vitro* findings demonstrating that SCEO effectively inhibits MDR *Salmonella* biofilm formation (Zhang et al., 2025), the present study extends this work by evaluating its *in vivo* efficacy. Since *Salmonella* infection typically originates in the gastrointestinal tract and may subsequently lead to systemic inflammation, an oral infection model was employed to better reflect physiological conditions and assess whether the *in vitro* anti-biofilm activity translates into *in vivo* protection.

Therefore, the aims of this study were to: (1) evaluate the *in vivo* protective efficacy and *in vitro* anti-inflammatory activity of SCEO; (2) identify its major bioactive constituents using GC–MS; and (3) predict potential targets through network pharmacology and molecular docking, followed by biological validation of the TLR4/NF-κB/MAPK signaling axis.

These findings may contribute to the development of plant-derived strategies for managing drug-resistant *Salmonella* infections and the associated inflammatory responses.

## 2 Materials and methods

### 2.1 Materials and reagents

The experimental animals were Kunming mice (18–22 g, 1:1 male-to-female ratio), purchased from Hunan Silek Jingda Laboratory Animal Co., Ltd. (via the Laboratory Animal Center of Guangxi University of Chinese Medicine). The MDR *Salmonella Thompson* isolate (C6304) used in this study was a well-characterized laboratory strain. It was previously isolated from aquatic food samples and screened for its high virulence and potent biofilm-forming ability, as detailed in our previous report (Zhang et al., 2025). Murine RAW264.7 macrophages were also maintained in our laboratory.

### 2.2 Mouse infection model and treatment protocol

Sixty specific pathogen free (SPF) Kunming mice were randomly assigned to six groups (n = 10/group): blank control, model, positive control (levofloxacin, 50 mg/kg/day), and three SCEO treatment groups (100, 200, and 400 mg/kg/day). To establish the intestinal infection model and overcome natural colonization resistance without utilizing antibiotic pre-treatment, mice were challenged via oral gavage with 0.2 mL/10 g of the *S. Thompson* suspension (1×10^9^ CFU/mL) for two consecutive days, while the blank control group received an equivalent volume of sterile saline. Successful infection was confirmed by the onset of diarrhea and retarded weight gain. Subsequently, treatments were administered via daily oral gavage for 7 consecutive days. The SCEO and positive control groups received their respective doses, whereas the blank and model groups were administered a 2% Tween-80 vehicle solution. Twelve hours after the final treatment, the mice were fasted (with *ad libitum* access to water), weighed, and anesthetized with 1.25% avertin. Blood and tissue samples were harvested. Livers and ileums were weighed to calculate organ indices, fixed in 4% paraformaldehyde, paraffin-embedded, sectioned, and stained with hematoxylin and eosin (HE) staining following standard histological protocols (Bancroft and Gamble, 2012).

### 2.3 Cell culture and cytotoxicity assay

RAW264.7 cells were seeded into 96-well plates (100 μL/well, 6 replicates/group). After attaching, the cells were treated with gradient concentrations of SCEO (0.001 to 1000 μg/mL) and incubated for 24 hours. The culture medium was then replaced with 100 μL of a DMEM and CCK-8 reagent mixture (10:1, v/v). Following a 2-hour incubation in the dark, absorbance was read at 450 nm (reference wavelength 630 nm) using a microplate reader to determine cell viability.

### 2.4 Active component identification and target prediction

To elucidate the bioactive constituents and potential therapeutic targets of SCEO against *Salmonella* infection, SCEO was prepared by blending CEO and SAEO at a synergistic ratio (fractional inhibitory concentration (FIC) = 0.375), as previously described (Zhang et al., 2025). Chemical profiling was performed using an SH-I-5 SIL MS capillary column (30 m × 0.25 mm i.d., 0.25 μm film thickness). The injection port temperature was maintained at 250 °C with a split ratio of 1:20. The oven temperature program was set as follows: initialized at 50 °C for 3 min, ramped to 140 °C at 5 °C/min and held for 5 min, increased to 180 °C at 5 °C/min and held for 5 min, and finally ramped to 280 °C at 10 °C/min and held for 3 min. Mass spectrometry was operated in electron ionization (EI) mode at 70 eV, with the ion source and interface temperatures set to 230 °C and 280 °C, respectively. Data were acquired in full-scan mode from *m/z* 40 to 1000 with a solvent delay of 3 min. Compound identification was achieved by matching mass spectra against the AROMA-5MS, AROMA-POLARWAX, NIST20-2, and NIST20S libraries. Detailed sample preparation and injection procedures are provided in Appendix **Supplementary Table 1**.

For the network pharmacology analysis, active compounds and their corresponding targets were retrieved from the Traditional Chinese Medicine Systems Pharmacology (TCMSP) and UniProt databases. Disease-related targets associated with “bacterial infection” and “*Salmonella* infection” were mined from the GeneCards database, applying a relevance score threshold of > 5. The intersection of SCEO compound targets and disease targets was visualized using Cytoscape (v3.7.2), and a protein-protein interaction (PPI) network was generated via the STRING database. Gene Ontology (GO) and Kyoto Encyclopedia of Genes and Genomes (KEGG) pathway enrichment analyses were performed using the MicroBioinformatics platform. Finally, the 3D co-crystal structures of core target proteins and small-molecule ligands were retrieved from the Protein Data Bank (PDB) and PubChem databases, respectively. Molecular docking was conducted on the CB-Dock2 platform and visualized with PyMOL.

### 2.5 Evaluation of inflammatory responses *in vitro* and *in vivo*

To assess the anti-inflammatory efficacy of SCEO, we quantified nitric oxide (NO) secretion and the mRNA expression of key inflammatory genes. RAW264.7 cells (5×10^4^ cells/well) were seeded into 48-well plates and allowed to adhere for 24 hours. The cells were then stimulated with 1 μg/mL lipopolysaccharide (LPS) and co-treated with SCEO at high (100 μg/mL), medium (50 μg/mL), or low (12.5 μg/mL) concentrations. A model group (LPS alone) and a blank group (culture medium alone) were included as controls. After a 20-hour incubation, NO levels in the culture supernatants were measured using a commercial NO assay kit (Catalog No. A013-2-1, Shanxi ShuoKe Biotechnology). For transcriptional analysis, total RNA was extracted from RAW264.7 cells and mouse liver tissues using the SPARKeasy Tissue/Cell RNA Rapid Extraction Kit, followed by reverse transcription into cDNA. Quantitative real-time PCR (qPCR) was performed with the 2×SYBR Green qPCR Mix on a 7500 Fast Real-Time PCR System (Applied Biosystems, USA). The specific primer sequences are provided in **Supplementary Table 2**. The relative mRNA expression levels of Toll-like receptor 4 (*Tlr4*), RELA proto-oncogene (*Rela*), mitogen-activated protein kinase 14 (*Mapk14*), interleukin-6 (*Il6*), tumor necrosis factor (*Tnf*), and interleukin-17A (*Il17a*) were normalized to glyceraldehyde-3-phosphate dehydrogenase (*Gapdh*) using the 2^(-ΔΔCt) method.

### 2.6 Statistical analysis

All statistical analyses and data visualizations were performed using SPSS 26.0 software (IBM Corp., Armonk, NY, USA). Differences between two groups were evaluated using the independent samples Student’s t-test, whereas multi-group comparisons were analyzed via one-way analysis of variance (ANOVA). A *P*-value < 0.05 was considered statistically significant.

## 3 Results

### 3.1 Protective effects of SCEO against Salmonella infection *in vivo*

**Figure 1:**
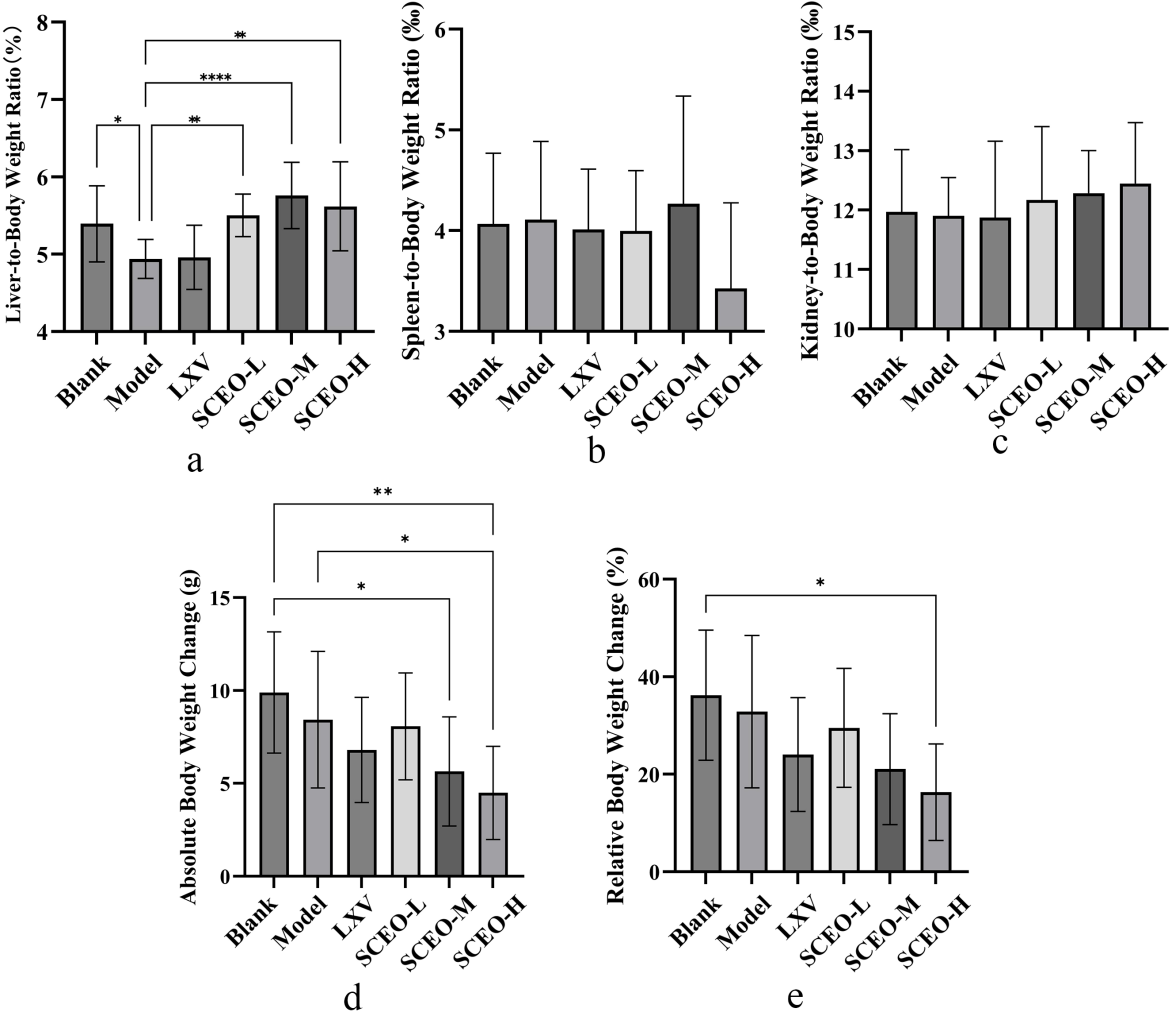
Changes in physiological indicators of Salmonella-infected mice after SCEO intervention. **(a)** Liver index; **(b)** Spleen index; **(c)** Kidney index; **(d)** Absolute body weight change value; **(e)** Relative body weight change value. * means a significant difference *P*<0.05, **means a highly significant difference *P*<0.01, ****means a highly significant difference *P*<0.001.

To evaluate the *in vivo* efficacy of SCEO, physiological parameters and histopathological changes in infected mice were assessed. Following *Salmonella* infection, the liver index in the model group was significantly reduced compared to the blank group (*P* < 0.05), indicating infection-induced hepatic atrophy. Conversely, the liver indices in all SCEO treatment groups (low, medium, and high doses) were significantly restored to near-normal levels (*P* < 0.05) (**Figure 1a**). No significant differences were observed in spleen and kidney indices across the groups (*P* > 0.05) (**Figures 1b, c**). Regarding body weight, the absolute and relative weight gains in the medium- and high-dose SCEO groups were significantly lower than those in the model group (*P* < 0.05).

**Figure 2:**
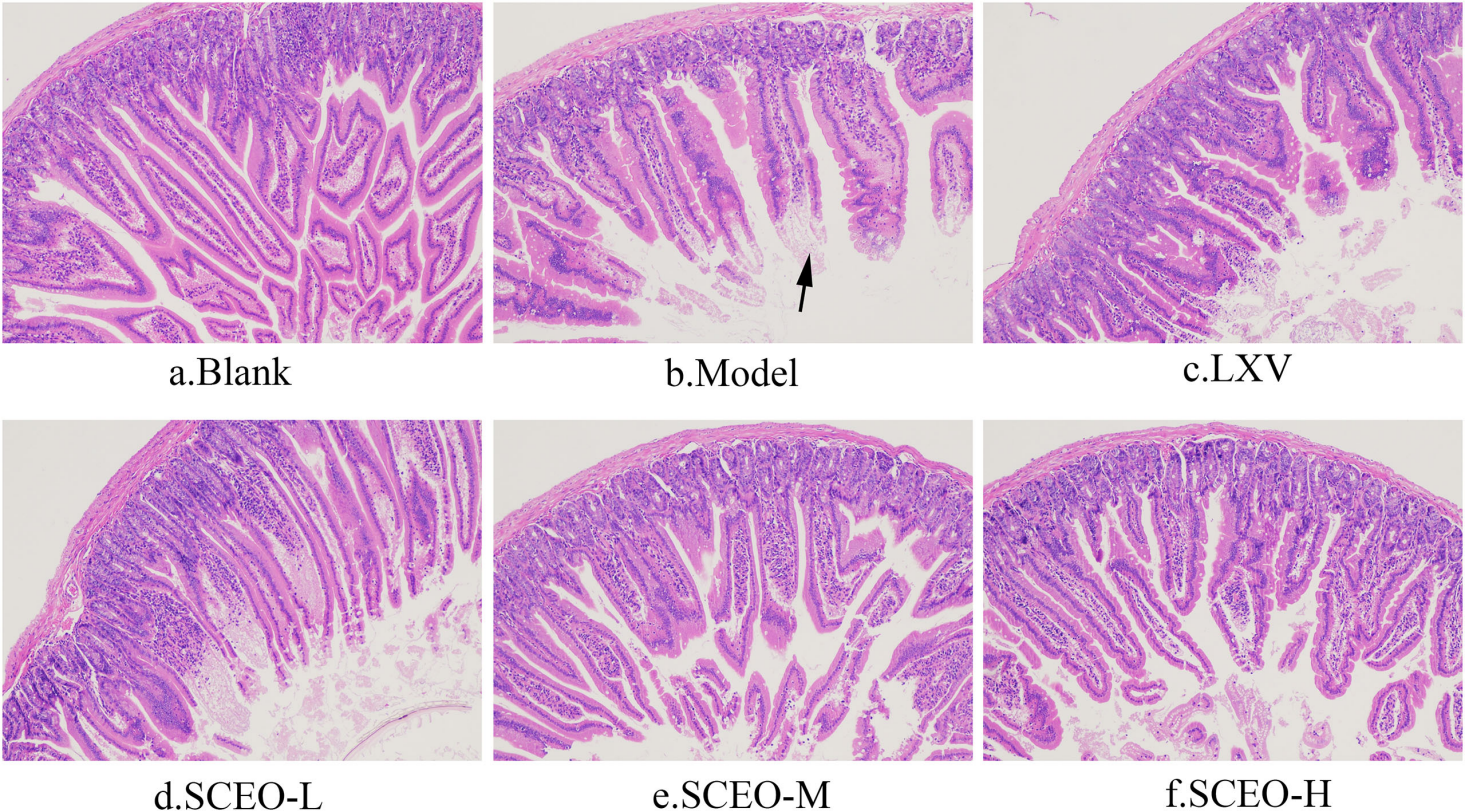
Six histological panels show stained mouse intestinal tissue sections labeled **(a–f)**. Panel **(a)** (Blank) displays healthy villi structure. Panel **(b)** (Model) shows disrupted villi with an arrow indicating damage. Panel **(c)** (LXV) and panels **(d–f)** (SCEO-L, SCEO-M, SCEO-H) present varying restoration of villi morphology.

**Figure 3:**
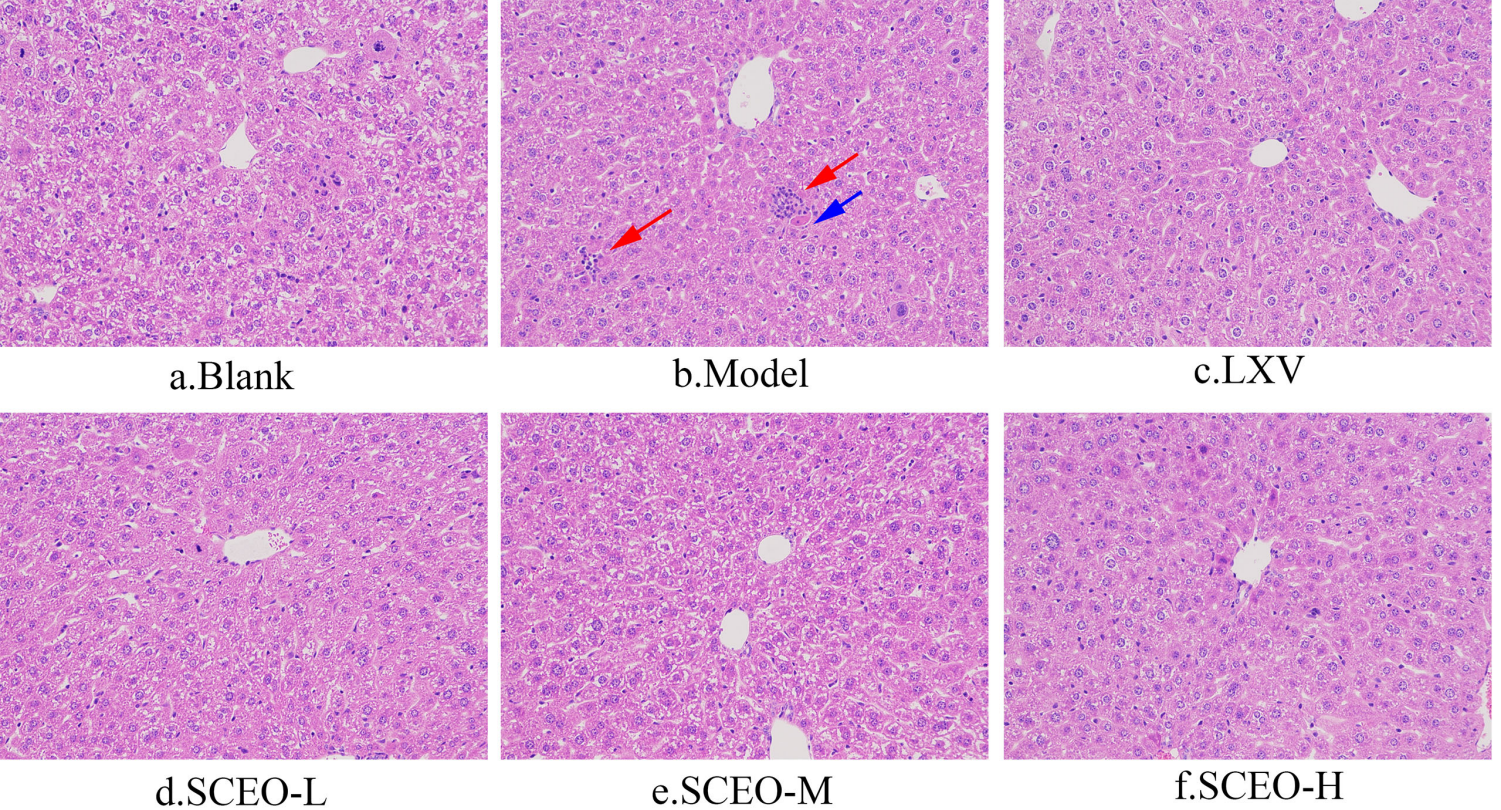
Microscope slide images display liver tissue stained with hematoxylin and eosin for six experimental groups labeled **(a–f)**. Group **(b)**, Model, indicates pathological changes with arrows highlighting cellular alterations compared to the healthier tissue structure in other groups.

Histopathological analysis (HE staining) further corroborated these observations. In the model group, intestinal villi exhibited structural irregularities, including shortening, thinning, severe inflammatory infiltration, and explicit partial shedding of the villus tips (indicated by the black arrow in **Figure 2b**). SCEO intervention dose-dependently ameliorated these damages; notably, the medium-dose group (**Figure 2e**) displayed the most intact villous architecture, closely resembling the blank control. Similarly, the liver tissues of the model group displayed disordered hepatocyte cords, distinct focal inflammatory cell infiltration (red arrows in **Figure 3b**), and hepatocyte degeneration such as nuclear pyknosis or vacuolization (blue arrow in **Figure 3b**). In contrast, SCEO treatment effectively preserved hepatic morphology. The hepatocytes in the SCEO-treated groups, particularly the medium-dose group (**Figure 3e**), were arranged orderly with clear nuclear-cytoplasmic boundaries and markedly reduced inflammatory foci, confirming the robust tissue-protective effect of SCEO.

### 3.2 Cytotoxicity and anti-inflammatory effects of SCEO *in vitro*

**Figure 4:**
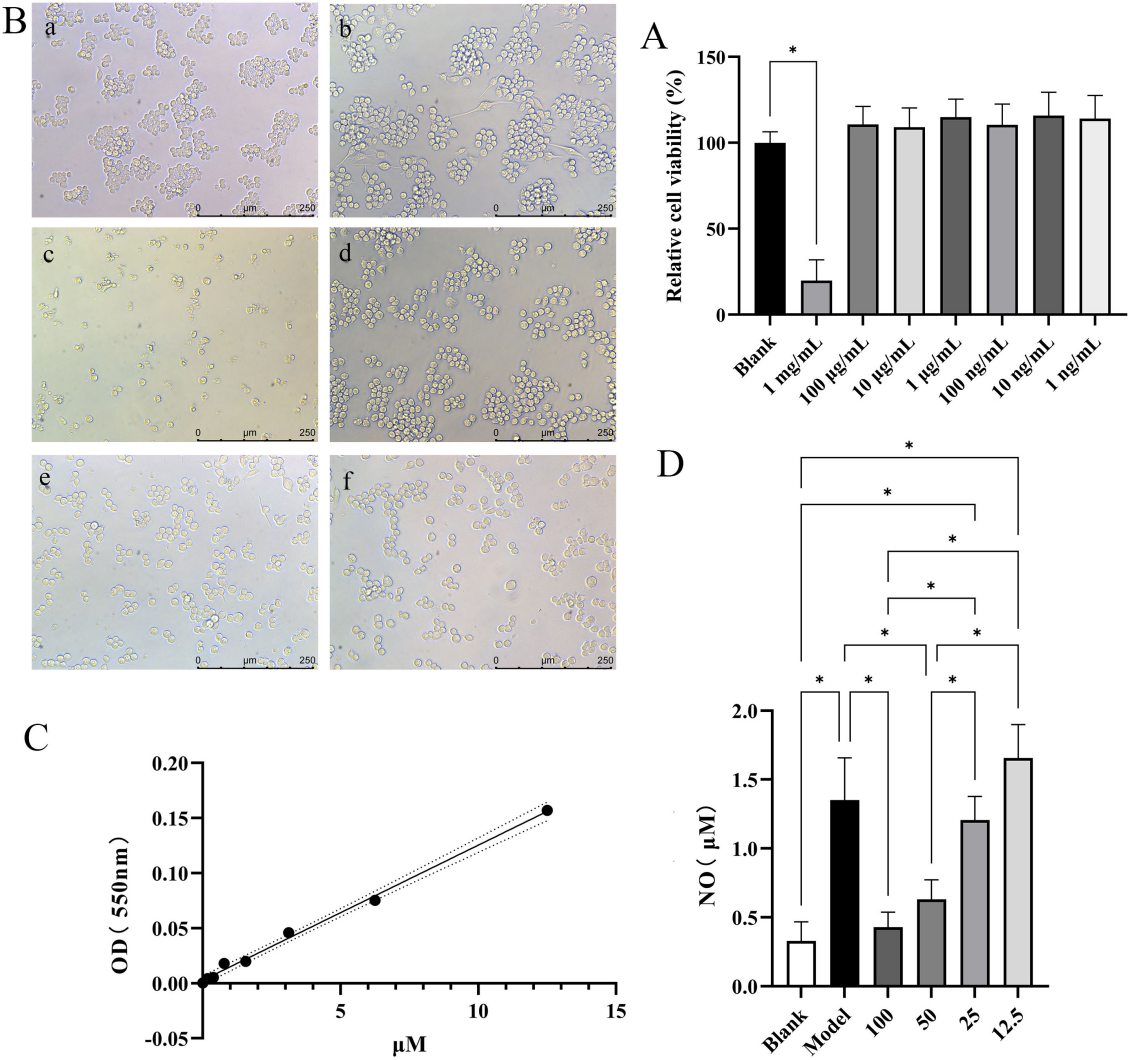
SCEO intervention in Salmonella LPS-induced cellular inflammation. **(A)** Relative viability of drug-treated cells detected by CCK-8 assay. **(B)** Microscopic images of RAW264.7 cells treated with LPS + gradient concentrations of SCEO. a (blank control group): untreated RAW264.7 cells; b (model group): LPS (1 μg/mL) alone; c-f (SCEO treatment group): LPS + gradient concentrations of SCEO (1 μL/mL, 100 nL/mL, 10 nL/mL, and 1 nL/mL). **(C)** NO standard curve, y = 0.0123x + 0.0438, R2 = 0.9952. **(D)** NO concentrations at different drug levels after SCEO treatment. * means a significant difference *P*<0.05.

To assess the safety profile and anti-inflammatory potential of SCEO *in vitro*, cell viability and NO secretion in RAW264.7 macrophages were evaluated. The CCK-8 assay revealed no significant cytotoxicity at SCEO concentrations ≤ 100 μg/mL, whereas 1000 μg/mL significantly reduced cell survival (**Figure 4A**). Morphologically, LPS stimulation induced severe cellular abnormalities and the presence of cell debris. However, treatment with 100 μg/mL SCEO remarkably reversed these LPS-induced changes, restoring uniform cell distribution and regular morphology with minimal pseudopodia (**Figure 4B**). Furthermore, SCEO exhibited a dose-dependent inhibitory effect on LPS-induced NO production. Concentrations of 50 and 100 μg/mL significantly suppressed NO secretion (*P* < 0.05) compared to the model group, highlighting its strong *in vitro* anti-inflammatory capacity (**Figure 4D**).

### 3.3 Identification of active components and target prediction

**Figure 5:**
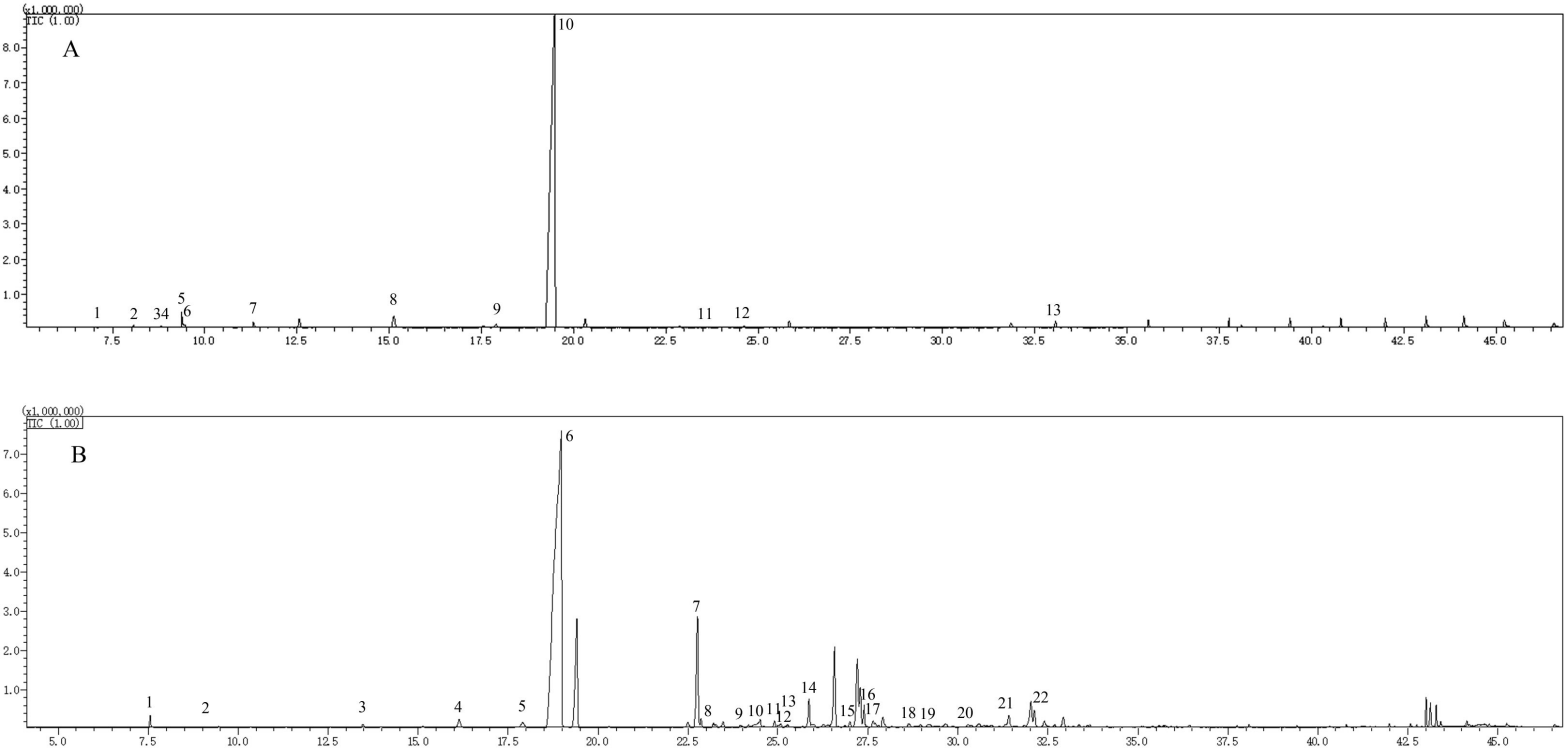
Total ion chromatograms of star anise and cinnamon essential oils by GC-MS. **(A)** shows the essential oil of star anise, with peaks 1–13 identified as: 1. α-pinene; 2. laurene; 3. α-norbornene (I); 4. δ-3-caryophyllene; 5. limonene; 6. α-norbornene (II); 7. eugenol; 8. eugenol (I); 9. 4-methoxybenzaldehyde; 10. trans-anethole; 11. β-caryophyllene; 12. cis-α-garantiene; 13.1-(3-methoxy-2-butenoxy)-4-(1-propenyl)benzene. **(B)** shows the essential oil of cassia bark, with peaks 1–22 identified as: 1. benzaldehyde; 2. benzyl alcohol; 3. benzaldehyde; 4. trans-cinnamaldehyde (I); 5. 4-methoxybenzaldehyde; 6. trans-cinnamaldehyde (II); 7. cubene; 8. 2-acetone, 1-(4-methoxyphenyl); 9. isofagopyrene; 10. cinnamic acid; 11. cassia bark acetate; 12. (Z)-2-methoxycassia bark aldehyde (I); 13. α-ligustrene; 14. α-erythrene; 15. trans-caragene; 16. (Z)-2-methoxycassia bark aldehyde (II); 17. α-caragene; 18. (E)-geranol; 19. caryophyllene alcohol; 20. longanone; 21. α-pinene; 22. τ-erythrene.

**Figure 6:**
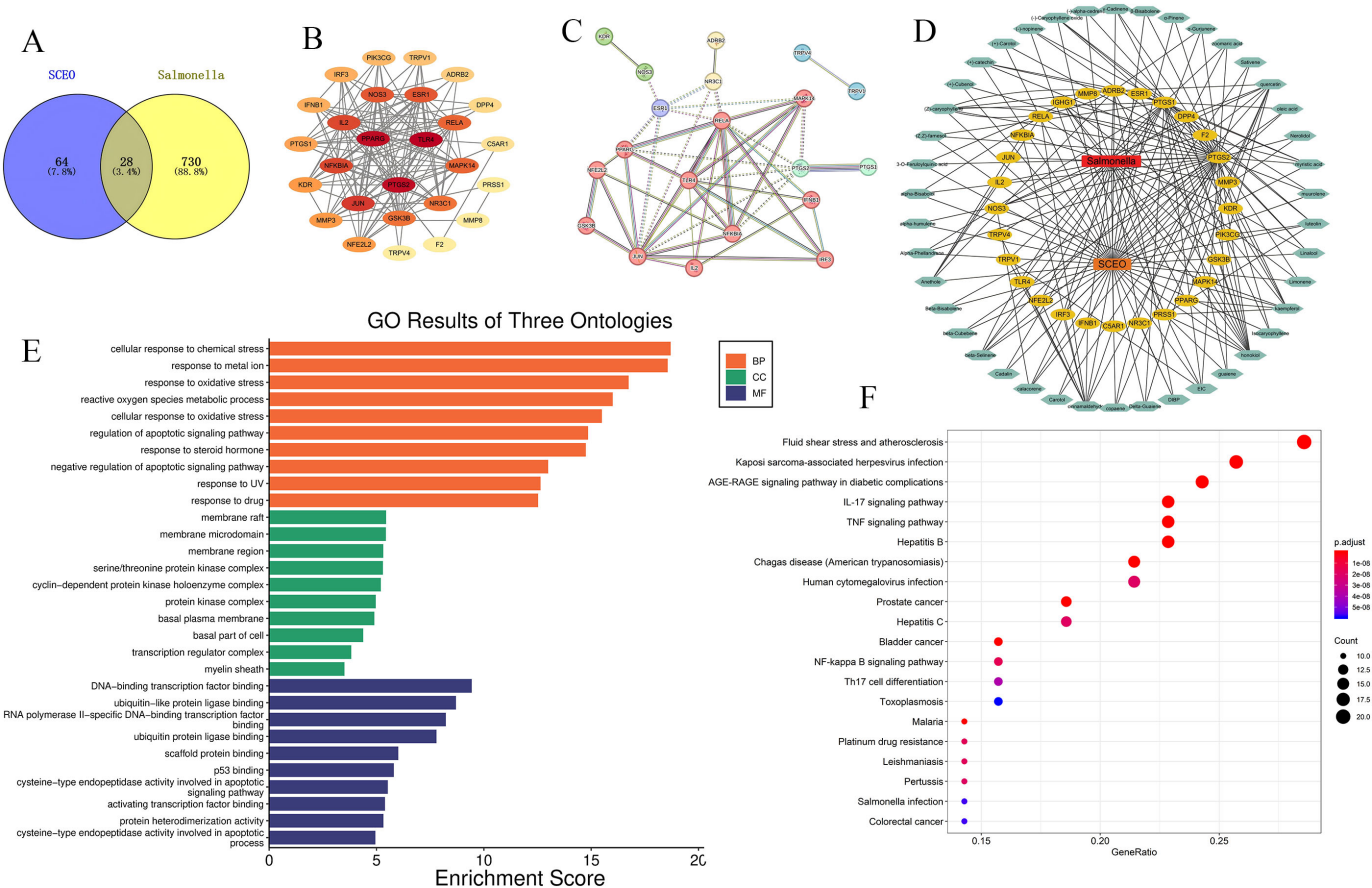
Network Pharmacology. **(A)** Venn diagram of drug-disease intersection targets. **(B)** PPI network diagram of potential target sites. **(C)** Functional modules identified by MCL clustering in the PPI network. Red (11 genes): Yersinia infection. Yellow (2 genes): ADRB2, NR3C1. Green (2 genes): KDR, NOS3. Light green (2 genes): Prostaglandin-endoperoxide synthase activity. Light blue (2 genes): Diet-induced thermogenesis. Blue (1 gene): ESR1. **(D)** Compound-target network diagram. **(E)** Gene Ontology (GO) enrichment analysis displaying top enriched terms in Biological Process (BP, orange), Cellular Component (CC, green), and Molecular Function (MF, purple) (enrichment score = -log10p). **(F)** Kyoto Encyclopedia of Genes and Genomes (KEGG) pathway enrichment analysis (dot plot); dot size represents gene count, and color intensity represents significance level (-log10 of adjusted p-value).

**Figure 7:**
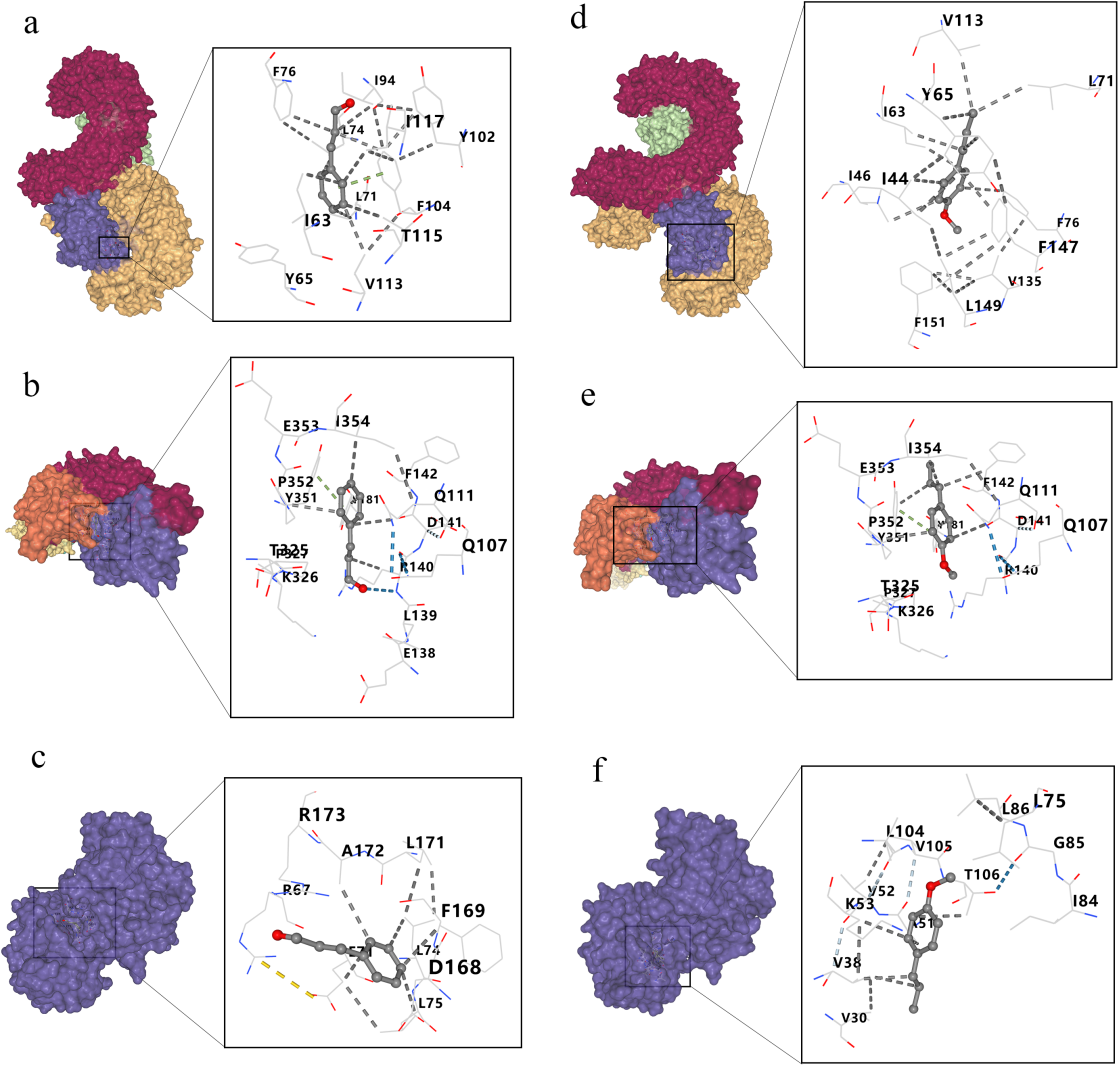
Molecular docking of trans-cinnamaldehyde and trans-anethole with RELA, TLR4, and MAPK. **(a)** trans-cinnamaldehyde docking with TLR4, vina = -5.9; **(b)** trans-anethole docking with TLR4, vina = -6.1; **(c)** trans-cinnamaldehyde docking with RELA, Vina = -6.1; **(d)** trans-anethole docking with RELA, Vina = -6.4; **(e)** trans-cinnamaldehyde docking with MAPK14, vina = -5.3; **(f)** trans-anethole docking with MAPK14, vina = -5.1.

To elucidate the material basis and potential therapeutic targets of SCEO, GC-MS, network pharmacology, and molecular docking were conducted. GC-MS analysis identified trans-cinnamaldehyde (74.49%) as the predominant component of CEO, followed by cubebene (7.36%) and α-caryophyllene (5.10%). In SAEO, trans-anethole was the absolute major constituent, accounting for 94.59% (**Figure 5**). Using network pharmacology, the intersection of 112 SCEO potential targets and 758 Salmonella infection-related targets yielded 28 core consensus targets (**Figure 6A**). The constructed Protein-Protein Interaction (PPI) network demonstrated tight functional modular synergy (average node degree = 3.41, local clustering coefficient = 0.523), far exceeding random network expectations (*P* < 1.0e-16). Key targets such as TLR4, RELA, and MAPK14 occupied core positions in this network (**Figure 6C**). GO and KEGG enrichment analyses revealed that these targets were primarily involved in response to oxidative stress, regulation of apoptotic signaling, and classical inflammatory pathways, notably the NF-κB, TNF, and IL-17 signaling pathways (**Figures 6E, F**). Furthermore, mapping these core targets onto the KEGG *Salmonella* infection pathway explicitly illustrated their critical positions within the TLR4-driven inflammatory cascade (**Supplementary Figure 1**). Molecular docking was subsequently performed to validate the binding affinities between the primary active components and core targets (**Figure 7**). Trans-cinnamaldehyde and trans-anethole exhibited strong binding to TLR4, RELA, and MAPK14 through robust hydrogen bonds and hydrophobic interactions (all Vina scores < -5.0 kcal/mol), theoretically confirming the feasibility of SCEO acting on this inflammatory axis.

### 3.4 SCEO modulates the TLR4/NF-κB/MAPK signaling axis

**Figure 8:**
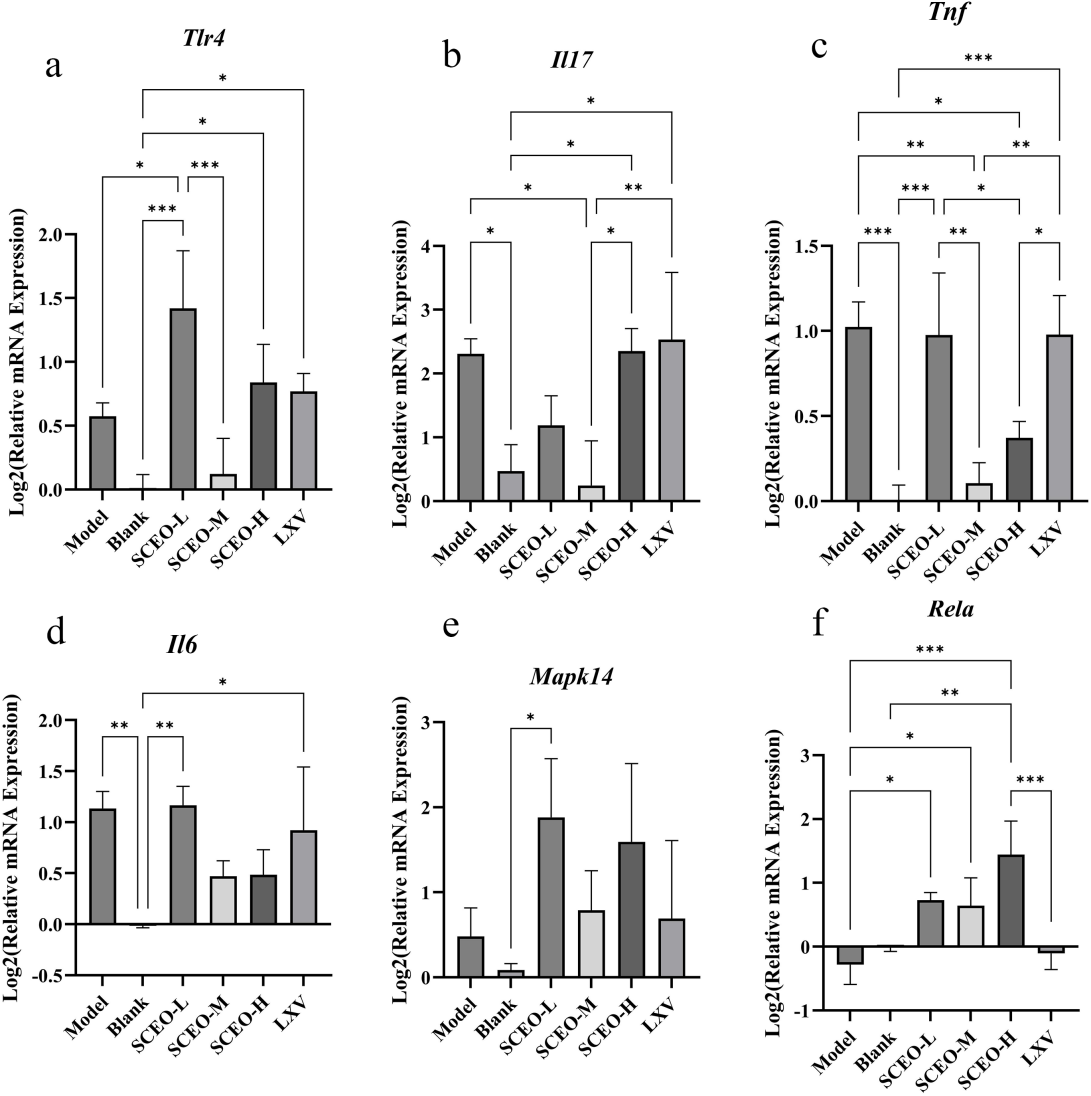
SCEO suppresses hepatic inflammatory signaling in *Salmonella*-infected mice. The mRNA expression levels of **(a)** *Tlr4*, **(b)** *Il17a*, **(c)** *Tnf*, **(d)** *Il6*, **(e)** *Mapk14*, and **(f)** *Rela* in liver tissues were quantified by qPCR. Data are expressed as Log2 relative to the blank control to accommodate the high magnitude of transcriptional changes. Mice were challenged with *S. Thompson* (Model) and treated with SCEO (100, 200, or 400 mg/kg) or levofloxacin (LXV, 50 mg/kg). Data are presented as mean ± SD (n = 3). *P < 0.05, **P < 0.01, ***P < 0.001 indicate statistical significance compared to the model group or between the indicated groups.

**Figure 9:**
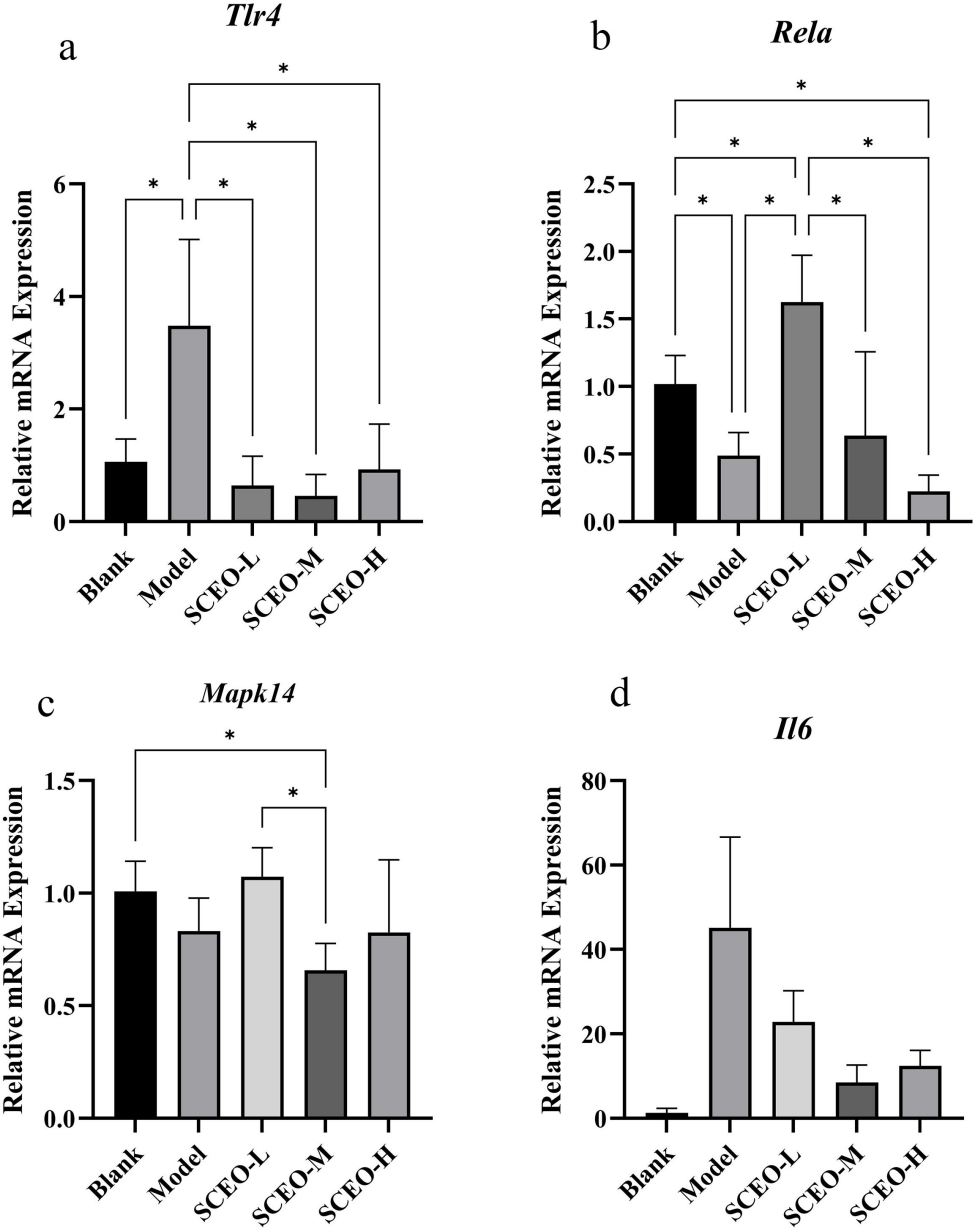
Four grouped bar graphs labeled **(a–d)** show relative mRNA expression for Tlr4, Rela, Mapk14, and Il6 across Blank, Model, SCEO-L, SCEO-M, and SCEO-H groups. Significant differences are indicated by asterisks above relevant comparisons.

To biologically validate the targets predicted by network pharmacology, the mRNA expression levels of key genes within the TLR4/NF-κB/MAPK pathway were quantified both *in vivo* and *in vitro*. In the mouse model, *Salmonella* infection significantly upregulated the hepatic mRNA expression of *Tlr4*, *Tnf*, *Il6*, and *Il17a* (*P* < 0.05) (**Figure 8**). Medium-dose SCEO intervention significantly suppressed the expression of *Tlr4*, *Tnf*, and *Il6* (*P* < 0.05), while also modulating the expression of *Mapk14* and *Rela*. These findings were highly consistent with the *in vitro* cellular model. In LPS-stimulated RAW264.7 cells, SCEO treatment dose-dependently downregulated the mRNA levels of *Tlr4* and *Il6* (*P* < 0.01)(**Figure 9**). Notably, high-dose SCEO effectively suppressed Rela expression (a 40% reduction compared to the model group, *P* < 0.01), and the medium dose notably reduced *Mapk14* transcription. Taken together, these results demonstrate that SCEO exerts its anti-inflammatory effects primarily by suppressing the TLR4/NF-κB/MAPK signaling axis and downregulating the transcription of downstream pro-inflammatory cytokines.

## 4 Discussion

In the face of escalating antimicrobial resistance, plant-derived essential oils have emerged as promising alternatives to conventional antibiotics. In this study, we demonstrated the significant protective efficacy of star anise-cinnamon compound essential oil (SCEO) against systemic inflammation induced by MDR *Salmonella Thompson*. By integrating GC-MS profiling, which identified *trans*-cinnamaldehyde and *trans*-anethole as the primary bioactive constituents (Ling et al., 2023; Quan, 2017), with *in vivo* and *in vitro* validations, our findings establish a solid material foundation for SCEO as a potent, multi-target immunomodulator.

*Salmonella* infection is characterized by severe systemic inflammation and target organ dysfunction, primarily triggered when the bacterial endotoxin (LPS) binds to the host TLR4 receptor (**Supplementary Figure 1**) (Ramm and Mally, 2013; Sargen and Helaine, 2024). Consistent with this pathogenesis, our *in vivo* model revealed that MDR *S. Thompson* infection caused pronounced hepatic atrophy, localized inflammatory infiltration, and severe intestinal villus shedding. Crucially, SCEO intervention effectively reversed these morphologic damages and normalized organ indices. This indicates that the synergistic action of the compound essential oil effectively bolsters the host’s physical and immune barriers against enteric bacterial invasion, extending the therapeutic potential beyond what is typically achieved by solitary essential oils (Huang et al., 2024; Saraswathy et al., 2010).

To map the molecular targets driving these protective phenotypes, we employed an integrated computational approach. Notably, applying conventional network pharmacology screening thresholds (e.g., oral bioavailability (OB) ≥ 30%, drug-likeness (DL) ≥ 0.18) may inadvertently omit abundant, highly volatile components like *trans*-cinnamaldehyde, a methodological constraint recently discussed in pharmacological research (Liao et al., 2024; Zheng et al., 2021). To address this limitation, we refined our selection criteria by utilizing high-proportion constituents validated by our GC-MS data. Through this approach, our topological analysis identified the TLR4/NF-κB/MAPK signaling axis as the core regulatory pathway, which was strongly corroborated by molecular docking.

Biologically, our *in vitro* and *in vivo* assays confirmed this computational prediction. *Trans*-anethole and *trans*-cinnamaldehyde exhibited high-affinity binding to TLR4 (Vina < -5.0 kcal/mol), theoretically obstructing LPS recognition. Correspondingly, SCEO dose-dependently downregulated *Tlr4* and *Il6* mRNA expression and significantly inhibited NO secretion in LPS-stimulated RAW264.7 macrophages. Previous independent studies have demonstrated that cinnamon extract specifically antagonizes TLR4 to suppress IL-8 in monocytes (Schink et al., 2018), and ameliorates LPS-induced acute lung injury via the TLR4/NF-κB pathway (Liu et al., 2024). Our data substantially expand upon this, proving that the SCEO complex similarly orchestrates robust anti-inflammatory responses against enteric *Salmonella* infections by directly disarming this classical inflammatory cascade.

Most intriguingly, our cellular assays revealed a nuanced, concentration-dependent divergence regarding NF-κB (RELA) regulation. High-dose SCEO significantly suppressed *Rela* expression, effectively halting excessive and damaging inflammatory storms. Conversely, low-dose treatment slightly upregulated its expression. We hypothesize that this low-dose activation might trigger protective autophagy or host antioxidant pathways to maintain basal immune homeostasis—a dynamic regulatory mechanism analogous to the oxidative stress modulation reported previously for cinnamaldehyde (Yin et al., 2022b). Furthermore, while trans-cinnamaldehyde bound effectively to MAPK14 in silico, *Mapk14* mRNA levels showed minimal fluctuation *in vitro*. This discrepancy implies that SCEO may modulate MAPK signaling primarily through post-translational modifications (e.g., inhibiting phosphorylation) rather than direct transcriptional suppression.

Building on our previous work demonstrating that SCEO inhibits *Salmonella* biofilm formation (Zhang et al., 2025), the present study extends these findings by further elucidating its anti-inflammatory effects in both *in vivo* and *in vitro* models. While the current results mainly emphasize the host-protective and tissue-preserving properties of SCEO, the absence of *in vivo* bacterial clearance data remains a limitation. Future studies should therefore aim to simultaneously assess bacterial burden in real time alongside these immunomodulatory effects. In addition, further investigations at the protein level (e.g., Western blot analysis) will be necessary to better understand its post-translational regulatory mechanisms. Nevertheless, our findings support the potential of SCEO as a promising natural therapeutic candidate for managing systemic inflammation caused by MDR *Salmonella.*

## 5 Conclusion

In summary, this study bridges the gap between *in vitro* antibacterial activity and *in vivo* therapeutic efficacy by systematically demonstrating the protective role of SCEO against MDR *Salmonella* infection. Beyond confirming its antimicrobial potential, our findings reveal that SCEO effectively alleviates intestinal and hepatic inflammatory damage through coordinated modulation of the TLR4/NF-κB/MAPK signaling axis.

By integrating chemical characterization, network pharmacology prediction, and experimental validation, this work delineates a multi-component, multi-target regulatory framework underlying the anti-inflammatory effects of SCEO. Collectively, these results not only provide mechanistic insight into the synergistic action of compound essential oils but also highlight their promise as a feasible plant-derived strategy for addressing refractory food-borne infections in the context of rising antimicrobial resistance.

## Data Availability

The datasets presented in this study can be found in online repositories. The names of the repository/repositories and accession number(s) can be found in the article/**Supplementary Material**.

## Supplementary material

Supplementary Figure 1 Signal transduction pathways associated with *Salmonella* infection.

Supplementary Table 1 GC-MS Sample Preparation and Injection Procedure.
