# Supplementary material for: Unveiling the anti-inflammatory power of star anise-cinnamon compound essential oil against MDR Salmonella
Source: Front Cell Infect Microbiol. 2026 Jul 13;16:1814361. doi: 10.3389/fcimb.2026.1814361 (PMC13403108; doi:10.3389/fcimb.2026.1814361)
Supplement: Supplementary Figure 1 — Signal transduction pathways associated with Salmonella infection. [file DataSheet1.docx]

**Table S1 GC-MS Sample Preparation and Injection Procedure**

| **Sample** | **concentration** | **Step** | **Volume of liquid** | **Volume of ethyl acetate** | **Dilution factor (single step)** | **Total dilution factor** |
| --- | --- | --- | --- | --- | --- | --- |
| CEO | 0.1 μL/mL | Step 1 | 10 μL of the original solution | 990 μL | 100× | 100× |
|  |  | Step 2 | Diluent for Step 1: 100 μL | 900 μL | 10× | 1000× |
|  |  | Step 3 | Diluent for Step 2: 100 μL | 900 μL | 10× | 10,000× |
| SAEO | 0.08 μL/mL | Step 1 | 10 μL of the original solution | 990 μL | 100× | 100× |
|  |  | Step 2 | Diluent for Step 1: 800 μL | 200 μL | 1.25× | 125× |
|  |  | Step 3 | Diluent for Step 2: 100 μL | 900 μL | 10× | 1250× |
|  |  | Step 4 | Diluent for Step 3: 100 μL | 900 μL | 10× | 12,500× |

**Table S2 Target gene Primer Information Table**

| **Target gene (mouse)** | **Primer direction** | **Sequence (5'→3')** |
| --- | --- | --- |
| *Tlr4* | Forward | AGCTCCTGACCTTGGTCTTG |
|  | Reverse | CGCAGGGGAACTCAATGAGG |
| *Rela* | Forward | TGCAACAAGGAATTTCTTGGTGG |
|  | Reverse | TGTGCAGTAGCAATCTGCTGT |
| *Mapk14* | Forward | CTGACCGACGACCACGTTC |
|  | Reverse | CTTCGTTCACAGCTAGGTTGC |
| *Il-6* | Forward | ACTTCCATCCAGTTGCCTTCTTGG |
|  | Reverse | TTAAGCCTCCGACTTGTGAAGTGG |
| *Tnf* | Forward | AGCCGATGGGTTGTACCTTG |
|  | Reverse | GTGGGTGAGGAGCACGTAGTC |
| *Il-17a* | Forward | CACAGTATGAGTGTCCTTCTG |
|  | Reverse | AGCTGTCTCCGTGTTTTAATCC |
| *Gapdh* | Forward | AGGTCGGTGTGAACGGATTTG |
|  | Reverse | TGTAGACCATGTAGTTGAGGTCA |


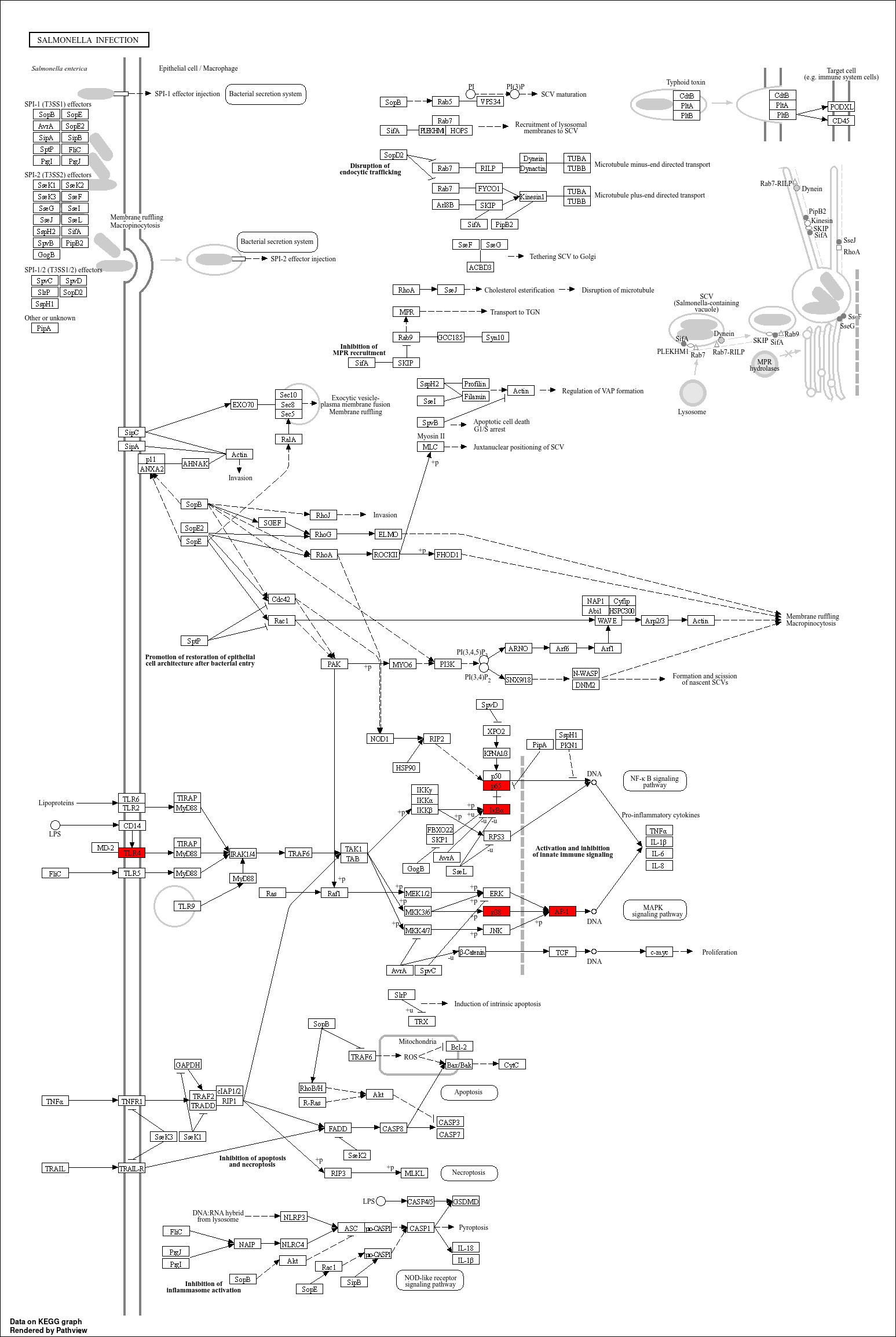
**Figure S1 Signal transduction pathways associated with *Salmonella* infection.**
